# OX40 Facilitates Control of a Persistent Virus Infection

**DOI:** 10.1371/journal.ppat.1002913

**Published:** 2012-09-06

**Authors:** Tobias Boettler, Friedrich Moeckel, Yang Cheng, Maximilian Heeg, Shahram Salek-Ardakani, Shane Crotty, Michael Croft, Matthias G. von Herrath

**Affiliations:** 1 Division of Developmental Immunology, La Jolla Institute for Allergy and Immunology, La Jolla, California, United States of America; 2 Division of Immune Regulation, La Jolla Institute for Allergy and Immunology, La Jolla, California, United States of America; 3 Division of Vaccine Discovery, La Jolla Institute for Allergy and Immunology, La Jolla, California, United States of America; Nationwide Children's Hospital, United States of America

## Abstract

During acute viral infections, clearance of the pathogen is followed by the contraction of the anti-viral T cell compartment. In contrast, T cell responses need to be maintained over a longer period of time during chronic viral infections in order to control viral replication and to avoid viral spreading. Much is known about inhibitory signals such as through PD-1 that limit T cell activity during chronic viral infection, but little is known about the stimulatory signals that allow maintenance of anti-viral T cells. Here, we show that the co-stimulatory molecule OX40 (CD134) is critically required in the context of persistent LCMV clone 13 infection. Anti-viral T cells express high levels of OX40 in the presence of their cognate antigen and T cells lacking the OX40 receptor fail to accumulate sufficiently. Moreover, the emergence of T cell dependent germinal center responses and LCMV-specific antibodies are severely impaired. Consequently, OX40-deficient mice fail to control LCMV clone 13 infection over time, highlighting the importance of this signaling pathway during persistent viral infection.

## Author Summary

A robust T cell response is the hallmark of an effective immune response to a variety of invading viruses. In many acute infections, the clearance of the viral pathogen is associated with a short and vigorous T cell response followed by development of pathogen-specific immune memory. However, some viruses can establish persistent infection in their respective host, during which an ongoing T cell response is required in order to prevent overwhelming viral replication. Little is known about the factors that sustain the T cell response in the persistent phase of a viral infection. In this report, we demonstrate that ligation of the OX40 molecule, which is expressed on T cells targeting the virus, is critically required in order to sustain the anti-viral immune response. We show that virus-specific, OX40-deficient T cells fail to accumulate sufficiently and consequently, mice lacking the OX40 receptor are incapable of controlling viral replication. Collectively our data establish OX40 as a crucial signaling molecule during a persistent viral infection.

## Introduction

Although persistent viral infections are typically associated with a dysfunctional and exhausted T cell signature [1], mice infected with the clone 13 (cl13) isolate of the lymphocytic choriomeningitis virus (LCMV) are able to control viral replication within 2–3 months post infection in a T cell dependent manner [2]–[7]. The inhibitory molecules involved in T cell exhaustion have been analyzed in great detail [1], [8]–[11]; yet, the signals that sustain T cell responses during persistent viral infections are not fully understood. This is a major concern, as, for example, the underlying cause for the loss of CD4 T cell responses during persistent viral infections in humans, such as chronic Hepatitis C Virus infection, is entirely unclear [12]. While recent studies demonstrated important roles for the Interleukins 21 and 6 in this context [2]–[4], [13], little is known about the role of co-stimulatory signals. OX40 (CD134) is a co-stimulatory molecule that has been shown to be important for T cell survival and function as well as establishment of T cell memory, although the degree to which OX40 influences immune responses is greatly context dependent [14]–[16]. While OX40 plays a role in driving T cell responses to several viruses [17]–[19], interestingly, it seems to be largely dispensable in the setting of acute LCMV infection. Although the LCMV-specific CD4 T cell responses in OX40-deficient mice are impaired, CD8 T cell responses, antibody titers and pathogen control are largely unaltered following acute LCMV infection [20]. However, the importance of OX40 during chronic viral infections remains unclear. Since OX40 signaling has the ability to promote long-term survival of T cells, we hypothesized that its biological relevance might be more prominent in the context of viral persistence. Indeed, in stark contrast to what has been observed during acute LCMV infection, we show that OX40 shapes both the CD4 and CD8 T cell response during persistent LCMV cl13 infection including T cell dependent antibody responses. The profound impact of OX40 expression in this context is highlighted by the observation that, in contrast to wild type mice, OX40-deficient mice are incapable of controlling viral replication.

## Results

### Strongly impaired anti-viral T cell responses in the absence of OX40

**Figure 1:**
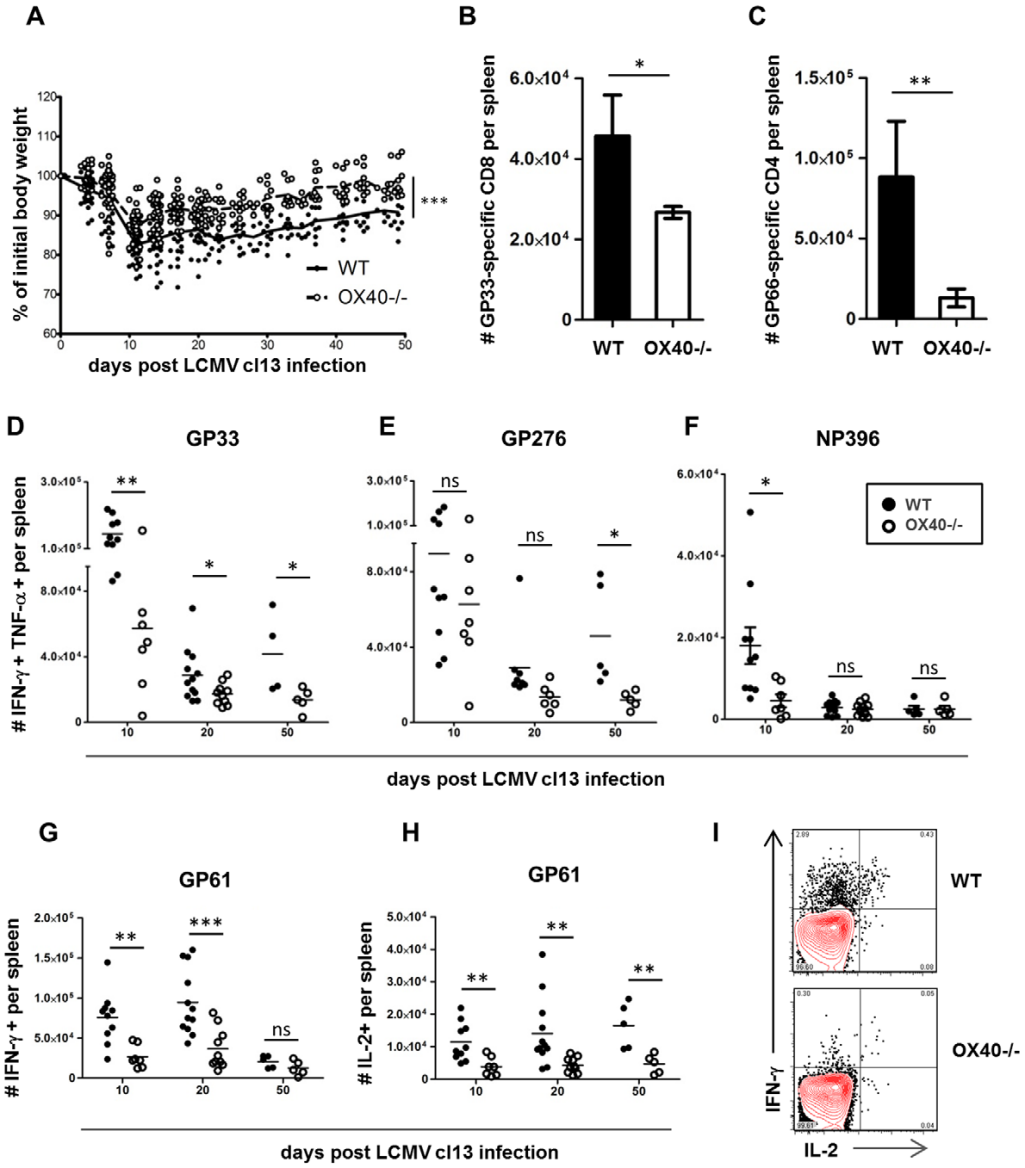
Impaired anti-viral CD4 and CD8 T cell responses in OX40−/− mice. WT (black circles) and OX40−/− (white circles) mice were intravenously infected with 2×10^6^ PFU of LCMV cl13. (A) Loss of body weight was measured twice weekly as a marker for disease severity. (B, C) Virus-specific splenic T cell populations in WT and OX40−/− mice were analyzed 20 days post infection using GP33-pentamers (B, CD8) and GP66-tetramers (C, CD4). (D–H) Splenocytes of infected mice were harvested 10, 20 and 50 days post infection. Intracellular cytokine stainings (ICCS) were performed following in vitro stimulation with immunodominant CD8 (D–F) and CD4 (G, H) epitopes as illustrated. (I) Representative ICCS staining following GP61 stimulation, gated on CD4 T cells. Data are derived from a total of 7 independent experiments, 1–2 experiments per time point.

Wild type (WT) and OX40-deficient mice (OX40−/−) on a C57BL/6 background were challenged intravenously with 2×10^6^ PFU of LCMV cl13 [21]. LCMV cl13 infection induces severe immunopathology in infected mice, particularly within the first two weeks post infection, characterized by excessive production of pro-inflammatory cytokines and extensive weight loss [22]–[24]. Interestingly, OX40−/− mice had a much healthier appearance and lost significantly less weight following cl13 infection (Fig. 1A). Next, we analyzed the impact of OX40 on the LCMV-specific T cell response. Using MHC class I and II restricted multimers, we detected significantly lower numbers of LCMV-specific CD8 and CD4 T cells in OX40−/− mice (Figure 1, B and C). The magnitude of the anti-viral CD4 and CD8 T cell responses over time was assessed by epitope-specific intracellular cytokine staining (ICCS), focusing on the production of the key effector and immunostimulatory cytokines IFN-γ, TNF and IL-2. In MHC H-2Db mice, the CD8 T cell response during LCMV cl13 infection is characterized by functionally impaired but sustained responses against two glycoprotein (GP) epitopes (GP33 and GP276) and a loss of T cells targeting the nucleoprotein (NP)-396 epitope. While the deletion of the NP396-specific response occurred regardless of the presence of OX40 (Fig. 1F), we constantly detected greater numbers of cytokine-producing cells in WT animals compared to OX40−/− mice in response to stimulation with the GP33 and the GP276 epitope (Fig. 1, D and E). The differences were even more pronounced in the anti-viral CD4 T cell compartment, where we detected significantly lower numbers of IFN-γ and IL-2 producing CD4 T cells in the absence of OX40 (Figure 1, G–I). These findings clearly suggest that OX40 ligation has a strong role in shaping the pro-inflammatory virus-specific T cell response during LCMV cl13 infection.

### Lack of germinal center responses in OX40-deficient mice

**Figure 2:**
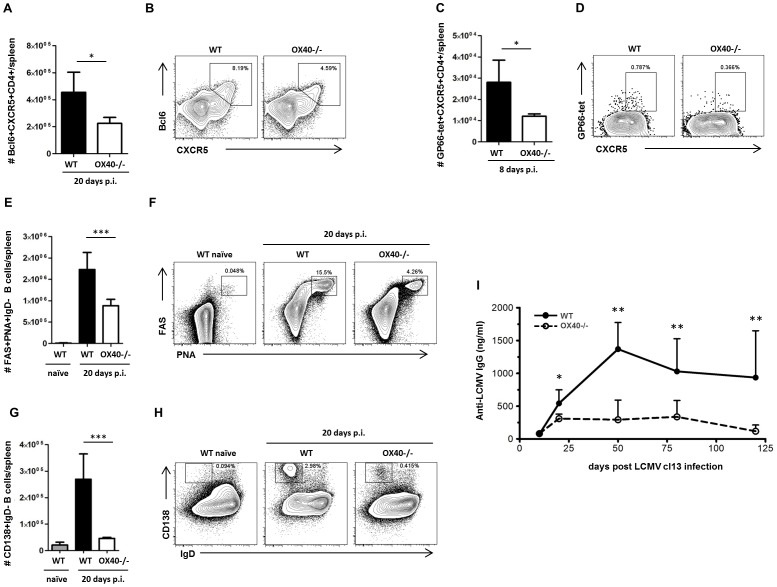
Impaired humoral immune response in OX40−/− mice. (A–H) Follicular T helper cell (Tfh) and germinal center responses were analyzed 8 respectively 20 days post LCMV cl13 infection in WT (black bars and lines) and OX40−/− mice (white bars and dashed lines) and compared to naïve WT mice (grey bars). (A) Number of Bcl6+CXCR5+ Tfh cells per spleen, (B) representative FACS-plots gated on CD4 T cells. (C) Number of LCMV-specific follicular helper CD4 T cells per spleen, (D) representative FACS-plots gated on CD4 T cells. (E) Number of germinal center B cells per spleen, (F) representative FACS-plots gated on B cells showing germinal center B cell populations. (G) Number of plasma cells per spleen, (H) representative FACS-plots gated on B cells showing plasma cell populations. Representative results from 1 of 2–3 independent experiments are illustrated. (I) LCMV-specific IgG antibody titers were analyzed by Elisa on days 10, 20, 50, 80 and 120 post infection (n = 4–10 per group and time point). Data are derived from a total of 7 independent experiments, 1–2 experiments per time point.

Next, we wanted to assess whether the disrupted T cell response in OX40−/− mice would also impact T cell help to B cells. CD4 T cell interactions with B cells are required for the development of germinal centers (GCs) and the emergence of antibody producing plasma cells [25], [26]. Follicular T helper cells (Tfh) represent the key CD4 T cell lineage associated with germinal center responses and are characterized by the expression of high levels of CXCR5 and PD-1 [25]. Two recent studies demonstrated that the absence of Tfh cells disabled LCMV-specific antibody production in cl13 infection, resulting in an inability of these mice to control viral replication [5], [13]. In OX40−/− mice, we detected significantly reduced numbers of LCMV-specific Tfh cells as characterized by the expression of CXCR5 (Fig. 2, A–D). Consequently, numbers of germinal center B cells (Fig. 2, E and F) as well as plasma cells (Fig. 2, G and H) were profoundly decreased in OX40−/− mice, resulting in significantly lower titers of LCMV-specific antibodies (Fig. 2I).

### OX40−/− mice fail to control LCMV cl13 infection

**Figure 3:**
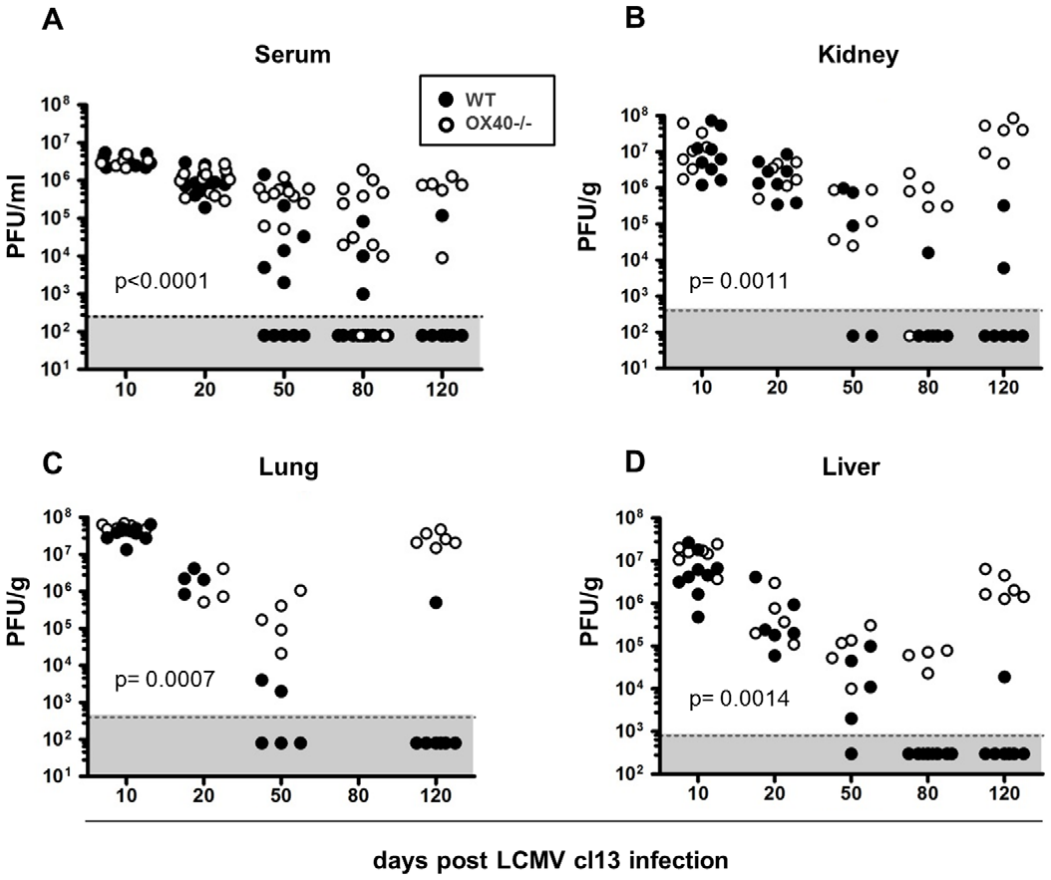
Lack of virus control in OX40−/− mice. (A) Serum, (B) kidney, (C) lung and (D) liver samples were harvested on days 10, 20, 50, 80 and 120 post infection and viral loads were determined by plaque assay. Statistical analysis for viral titers was performed using log rank test, undetectable viral titers were defined as endpoint. Data are derived from a total of 8 independent experiments, 1–2 experiments per time point.

While OX40−/− mice benefited clinically from the impaired T cell responses, as they lost markedly less weight than WT mice (Fig. 1A), we analyzed how this would affect their capability to control viral replication. To address this question, we quantified viral titers at various time points post infection in the serum and several solid organs by plaque assay. Interestingly, we found that OX40−/− and WT mice had very similar viral titers on days 10 and 20 post infection in most tissues (Fig. 3, A–D). However, while WT mice were able to control viral titers as soon as 50 days post infection in selected tissues such as the lung and serum, OX40−/− mice failed to control the virus to a similar degree. This difference was even more striking by days 80 and 120 post infection, when viral titers dropped below detection levels in most tissues in WT mice, whereas the majority of OX40−/− displayed high titers in most organs and serum (Fig. 3, A–D).

### OX40 is differentially expressed during acute and persistent LCMV infection

**Figure 4:**
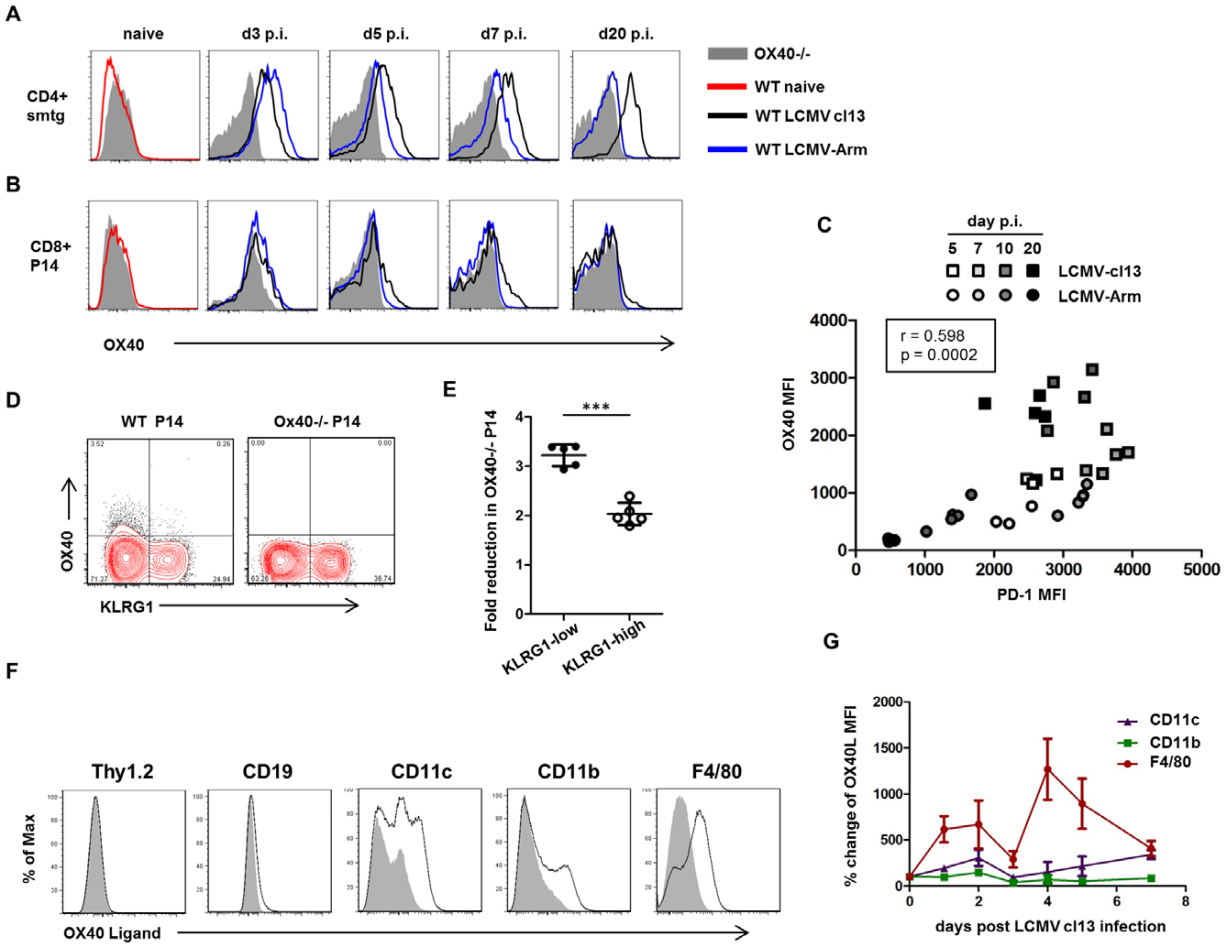
OX40 and OX40L expression during LCMV infection. (A, B) Naïve GP61-specific TCRtg CD4 T cells (smarta, smtg) and GP33-specific TCRtg CD8 T cells (P14) were transferred into WT recipients prior to acute and persistent LCMV infection. OX40 expression on both populations was analyzed before and 3, 5, 7 and 20 days post infection by flow cytometry. (C) PD-1 and OX40 expression was analyzed on virus-specific CD4 T cells (smarta, smtg) 5, 7, 10 and 20 days post LCMV Armstrong and cl13 infection. Data are derived from a total of 7 independent experiments, 1–2 experiments per time point. (D) Equal numbers of CD45.1/2 mismatched WT and OX40−/− P14 cells were co-transferred into WT recipients prior to LCMV cl13 infection. Expression of OX40 and KLRG1 was determined on both populations 8 days post LCMV cl13 infection. FACS-plot gated on P14 T cells. (E) Fold reduction of the OX40−/− P14 compared to WT P14 in the KLRG1-low and KLRG1-high gate was analyzed 8 days post LCMV cl13 infection. Data are derived from a total of 2 independent experiments. (F) Expression of OX40 Ligand was determined by flow cytometry 5 days post infection on T cells (Thy1.2), B cells (CD19) and antigen presenting cell populations (CD11c+Thy1.2-CD19−; CD11b+Thy1.2-CD19−; F4/80+Thy1.2-CD19−) following collagenase digestion. Data are derived from a total of 2 independent experiments. (G and H) Changes in the OX40L expression levels on the same antigen presenting cell populations were examined following LCMV cl13 infection. Isotype MFI was subtracted from OX40L MFI and plotted in relation to expression levels on naïve cells. Time course experiment was performed once.

To analyze the OX40 expression kinetics on virus-specific CD4 and CD8 T cells during acute and persistent LCMV infection, we transferred naïve, congenically marked TCR transgenic (TCRtg) CD4 (smarta, smtg) and CD8 (P14) T cells into WT recipients and analyzed OX40 expression on days 3, 5, 7, and 20 post LCMV Armstrong or LCMV cl13 infection. While OX40 was not expressed on naïve P14 and smtg cells, we found that it was strongly induced on both populations 3 days following acute and persistent LCMV infection (Fig. 4, A and B). Interestingly, OX40 expression levels on both CD4 and CD8 T cells rapidly declined in acutely infected mice but were maintained in persistently infected mice. In order to analyze whether the sustained OX40 expression during persistent LCMV infection could be related to ongoing antigen recognition, we simultaneously analyzed PD-1 and OX40 expression on virus-specific T cells, since PD-1 expression has been shown to be directly linked to antigen-expression in this system [27]. Interestingly, PD-1 expression strongly correlated with OX40 expression during both acute and persistent LCMV infection suggesting that the sustained OX40 expression during LCMV cl13 infection is a consequence of ongoing antigen recognition (Fig. 4C) and in line with prior data showing that TCR signaling is sufficient to induce OX40 on T cells [28]. Although OX40 receptor expression was stronger on CD4 T cells, a subset of antiviral CD8 T cells also displayed sustained OX40 expression. Thus, we wanted to examine on which subpopulation within the antiviral CD8 T cells OX40 expression might be most relevant. Therefore, we looked at the expression of the inhibitory killer cell lectin-like receptor G1 (KLRG1) on CD8 T cells which has been associated with a short-lived effector phenotype [29], [30] and has previously been linked to OX40 [31]. Interestingly, we found that OX40 expression on CD8 T cells is largely restricted to the KLRG1-low compartment during LCMV cl13 infection (Fig. 4D), suggesting that OX40 preferentially acts to maintain higher numbers of these longer-lived effector T cells during the persistent phase of infection. Indeed, the analysis of the KLRG1-low and the KLRG1-high CD8 compartments revealed that the absence of OX40 had a significantly stronger impact on the accumulation of KLRG1-low P14 cells compared to KLRG1-high P14 cells (Fig. 4E).

Next, we analyzed which cells could be capable of engaging the OX40 receptor through expression of the OX40 ligand (OX40L). OX40L is the only ligand that is known to activate the OX40 receptor and is typically expressed on activated antigen presenting cells, but also has been visualized on lymphoid tissue inducer (LTi) cells and activated/inflamed endothelium [15]. First, we analyzed OX40L expression on splenocytes that were harvested 5 days post LCMV cl13 infection. Not surprisingly, OX40L expression was largely limited to professional antigen presenting cells, slightly detectable on B cells and almost absent on T cells (Fig. 4F). Next, we wanted to analyze the dynamics of OX40L expression following LCMV cl13 infection. Therefore we harvested and stained splenocytes from uninfected and LCMV cl13 infected mice on defined days post infection. A precise phenotypic analysis of antigen presenting cell subsets over time is very challenging in the LCMV cl13 model as those cells can dramatically change their appearance due to immune activation and direct infection of those cells. Thus, we analyzed OX40L expression independently on bulk CD11c+, CD11b+ and F4/80+ cells after exclusion of T and B cells. Interestingly, we observed that OX40L was strongly upregulated on F4/80 expressing cells following LCMV cl13 infection, whereas it was largely unaltered in CD11b and CD11c expressing cells (Fig. 4G). Given that the true extent of OX40 ligand expression is difficult to assess due to several modes of regulation typical in the TNF superfamily, including activation-induced cleavage, receptor-induced cleavage, and rapid turnover and internalization, these data suggest that macrophage/dendritic lineage cells may provide the ligand for T cell expressed OX40 but do not rule out other cell types as possible sources.

### OX40 is required for accumulation of virus-specific T cells

**Figure 5:**
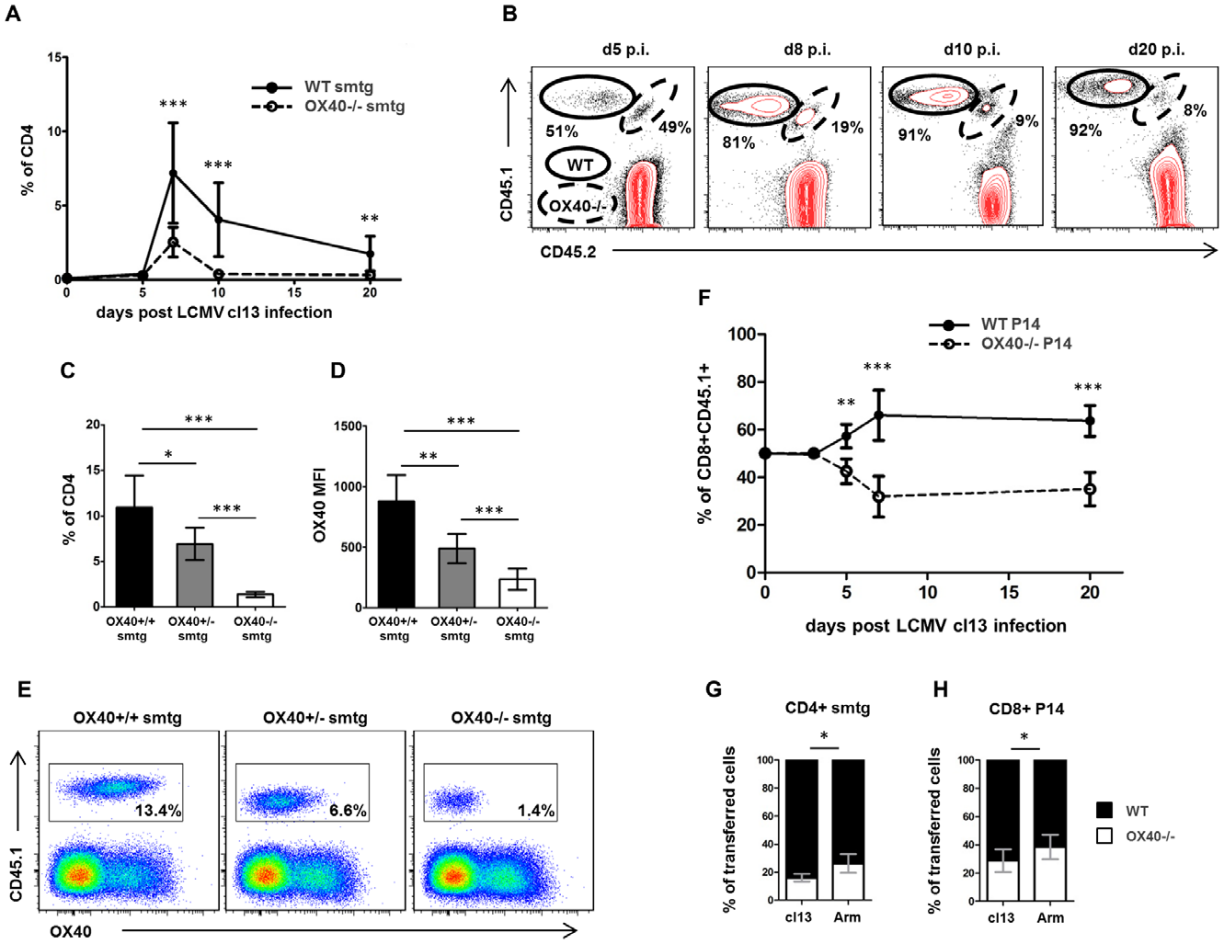
OX40 promotes T cell accumulation in a dose dependent manner. (A, B) Equal numbers of WT (CD45.1+CD45.2−) and OX40−/− (CD45.1+CD45.2+) smtg were transferred into the same WT recipients (CD45.1−CD45.2+) prior to LCMV cl13 infection. Splenocytes were harvested on days 5, 8, 10 and 20 post infection (n = 4–8 for each time point) and (A) frequencies of OX40−/− smtg and WT smtg were determined within the CD4 gate by flow cytometry. (B) Frequencies shown in FACS-plots are percent of CD45.1+ (transferred cells); plots are gated on CD4 T cells. (C–E) OX40+/+ (WT, black bars), OX40+/− (Het, grey bars) and OX40−/− (KO, white bars) smtg were separately transferred into WT recipients prior to LCMV cl13 infection and (C) frequencies of WT, Het and KO smtg cells within the CD4 gate were determined 10 days post infection (n = 5 per group). (D) OX40 expression on WT, Het and KO smtg cells was determined 10 days post infection by flow cytometry (n = 5 per group). (E) Representative FACS-plots showing transferred cells and OX40 expression gated on CD4 T cells. (F) Equal numbers of CD45.1/2 mismatched WT and OX40−/− P14 cells were co-transferred into WT recipients prior to LCMV cl13 infection and frequencies of OX40−/− P14 and WT P14 were determined within the transferred P14 population by flow cytometry on day 3, 5, 8 and 20 post infection (n = 4–5 for each time point). (G, H) Similar co-transfer experiments with CD4 and CD8 T cell were performed in order to compare the role of OX40 on a single cell level in acute and chronic LCMV infection. Graph shows percentage of transferred OX40−/− CD4 respectively OX40−/− CD8 T cells of all transferred T-Cells 8 days post infection. Data are derived from 2–3 independent CD4 smtg cell transfer experiments per time point and 1–3 independent CD8 P14 cell transfer experiments per time point.

To assess the direct impact of OX40 expression on virus-specific CD4 and CD8 T cells, we crossed TCRtg CD4 (smtg) and CD8 (P14) T cells onto the OX40−/− background. We then co-transferred equal numbers of naïve WT (CD45.1+CD45.2−) and OX40−/− (CD45.1+CD45.2+) cells into C57BL/6 (CD45.1−CD45.2+) recipients prior to LCMV cl13 infection or LCMV Armstrong infection. Strikingly, WT CD4 smtg cells accumulated to much higher numbers compared to OX40−/− smtg cells (Fig. 5A). The number of WT smtg cells within the transferred population was approximately 4 fold increased compared to OX40−/− smtg cells as soon as day 8 p.i. (Fig. 5B). Following the initial contraction phase around day 10 p.i. this difference was approximately 10 fold (Fig. 5B). On day 20 p.i, the OX40−/− smtg compartment was almost entirely lost (Fig. 5, A and B). To address whether OX40 signals are dose dependent in this setting, we transferred equal numbers of WT (OX40+/+), heterozygous (OX40+/−) and OX40-deficient (OX40−/−) smtg cells into separate WT hosts. Indeed, OX40+/+ smtg cells expressing the highest levels of OX40 following LCMV cl13 infection accumulated significantly better than OX40+/− smtg cells and OX40−/− smtg cells (Fig. 5, C–E). When we performed similar co-transfer experiments with WT and OX40−/− CD8 TCRtg P14 cells, consistent with our findings on the CD4 level, we repeatedly harvested higher numbers of WT P14 cells compared OX40−/− P14 cells from spleens of persistently infected mice (Fig. 5F).

In order to directly compare the role of OX40 during acute and persistent LCMV infection, we performed co-transfer experiments with WT and OX40−/− TCRtg CD4 and CD8 T cells prior to LCMV Armstrong and cl13 infection. In agreement with the observation of reduced OX40 receptor expression during acute infection (Fig. 4, A and B), the percentage of OX40−/− CD4 and CD8 TCRtg cells within the ‘transferred cells’ gate was significantly lower in cl13 infected animals than in Armstrong infected animals (Fig. 5, G and H).

### OX40 does not directly impact antiviral T cell function but promotes T cell survival by modulating Bcl-2 and Bcl-xL expression

**Figure 6:**
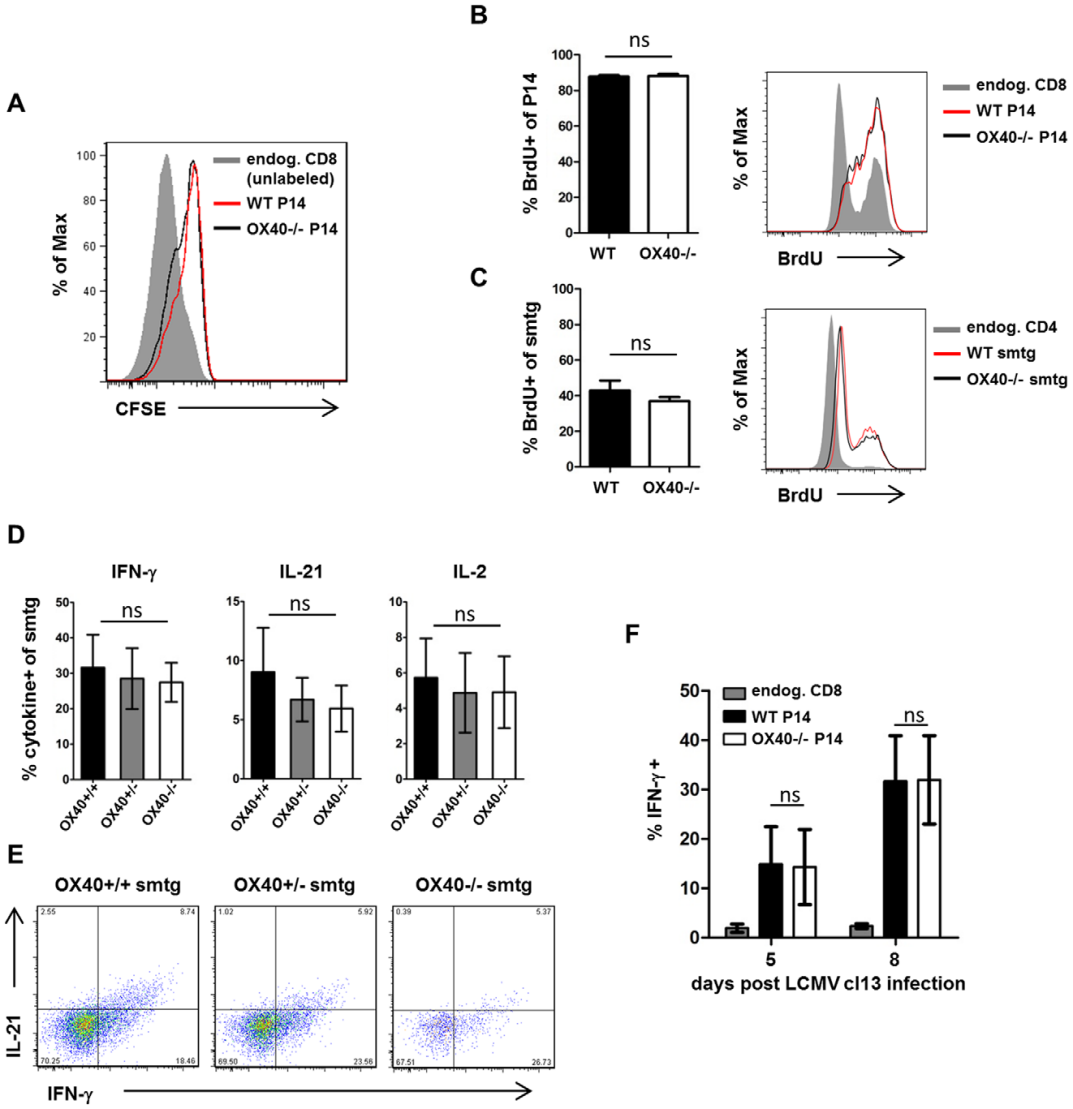
OX40 is dispensable for T cell proliferation and direct antiviral T cell function. (A) WT and OX40−/− P14 cells were labeled with CFSE prior to injection in WT recipients. Graph is showing CFSE dilution of congenically marked TCR transgenic (WT red, OX40−/− black) and unlabeled endogenous CD8 T cells (grey area) 5 days post infection. (B, C) CD45.1/2 mismatched WT and OX40−/− TCRtg cells were co-transferred into WT recipients prior to LCMV cl13 infection and BrdU was injected intraperitoneally on day 6 post infection according to the manufacturer's instructions. Cells were harvested and BrdU staining was detected by flow cytometry to visualize cells that had proliferated. Data are representative for 1 out of 2 independent experiments. (D) Intracellular cytokine staining (ICCS) for IFN-γ, IL-21 and IL-2 was performed 10 days post infection following peptide stimulation (n = 5 per group). (E) Representative FACS-plots showing IFN-γ and IL-21 production of WT, Het and KO smtg cells. (F) Intracellular cytokine staining (ICCS) for IFN-γ following GP33 peptide stimulation on congenically marked TCR transgenic CD8 T cells (P14) transferred into WT recipients was performed 5 and 8 days post infection (n = 4–5 per group). Data are derived from 2 independent CD4 smtg cell transfer experiments and 2 independent CD8 P14 cell transfer experiments.

**Figure 7:**
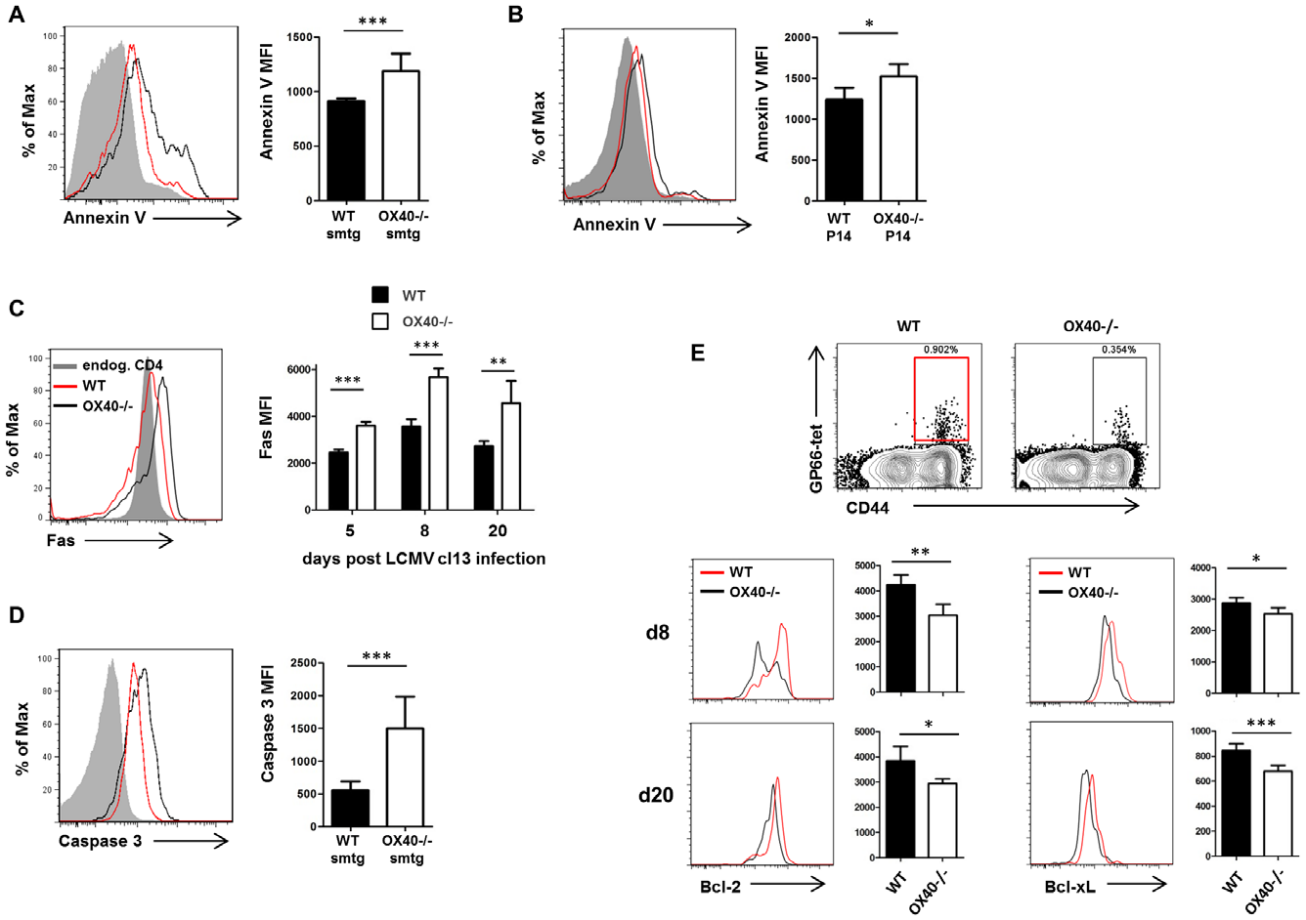
OX40 regulates T cell survival and Bcl-2 and Bcl-xL. (A–D) Fas, Annexin V and active caspase 3 analyses were performed to detect apoptotic signals in co-transferred TCRtg cells (WT red, OX40−/− black) and endogenous CD4 and CD8 T cells (grey area) after LCMV cl13 infection. (A, B) Representative Annexin V stainings and bar graphs showing Annexin V MFI on CD4 smtg cells on day 8 p.i. (A) and on CD8 P14 cells on day 10 p.i. (B). (C) Representative Fas staining on day 8 p.i. and bar graph showing Fas expression 5, 8, and 20 days post infection on CD4 smtg cells. (D) Representative intracellular staining for active caspase 3 and bar graph showing Caspase 3 MFI on day 8 p.i. on CD4 smtg cells. Data are derived from 3 independent CD4 smtg cell transfer experiments and one CD8 P14 transfer experiment. (E) Analysis of expression of anti-apoptotic molecules Bcl-2 and Bcl-xL on virus-specific CD4 T cells of WT and OX40−/− mice (WT red, OX40−/− black) via intracellular protein staining 8 and 20 days post persistent LCMV infection. Data are derived from 1–2 independent experiments per time point.

Signals through OX40 can influence several aspects of T cell biology, such as survival, proliferative capacities and the ability to secrete cytokines [15]. In order to define the precise role of OX40 on antiviral T cells during persistent infection, we first examined the functional capacities of WT and OX40-deficient cells in co-transfer experiments. We performed CFSE- and BrdU- labeling experiments to study the ability of WT and OX40−/− CD4 and CD8 TCRtg cells to proliferate. Those experiments revealed that T cell proliferation occurred independently of OX40 signals over the initial course of infection (Fig. 6, A–C), demonstrating that the lack of accumulation is unlikely to be a consequence of impaired cell division in this model. Secondly, we analyzed cytokine production of antiviral T cells since we found drastically reduced numbers of cytokine producing CD4 and CD8 T cells in OX40−/− mice (Fig. 1, D–I). Analysis of IL-2, IL-21 and IFN-γsecretion by WT, Het and KO smtg cells and IFN- γ secretion by WT and OX40−/− P14 cells revealed modest increases in the percentage of cytokine producing cells in the WT compared to Het and KO TCRtg T cells. However, these differences failed to consistently reach statistical significance, suggesting that OX40 signals are not required for direct anti-viral T cell function (Fig. 6, D and E). Similarly, we did not detect differences in IFN- γ secretion by WT and OX40−/− P14 cells (Fig. 6F). Lastly, we investigated whether OX40−/− cells were more prone to apoptosis, since OX40 has been shown to antagonize apoptotic cell death in T cells [14], [15]. Indeed, OX40-deficient T cells displayed significantly stronger Annexin V binding, suggestive of a higher number of apoptotic cells within the OX40-deficient compartment (Fig. 7, A and B). To examine what pathways might be involved, we focused on CD4 T cells and found significantly elevated levels of the pro-apoptotic Fas-receptor on days 5, 8 and 20 post infection on OX40-deficient cells (Fig. 7C) suggestive of negative regulation of Fas by OX40. Moreover, this was associated with higher levels of active caspase 3 in those cells (Fig. 7D). OX40 has also been shown to upregulate anti-apoptotic Bcl-2 family members, such as Bcl-2 and Bcl-xL in T cells [14]. OX40-deficient T cells fail to maintain Bcl-2/Bcl-xL levels and showing the significance of this, retrovirally introducing those proteins back into OX40-deficient CD4 and CD8 T cells restores their survival capacities [14], [32]. Expression of Bcl-2 family members is directly dependent on OX40 mediated NF-κB1 and Akt activity as OX40 deficient T cells display reduced activation of both NF-κB1 and Akt and restoring these activities by introducing active IκB kinase or active Akt rescues survival and Bcl-2/Bcl-xL expression in OX40 deficient T cells [33]–[35]. Indeed, when analyzing Bcl-2 and Bcl-xL expression on virus-specific CD4 T cells by tetramer and intracellular antibody-staining, we found significantly higher levels of Bcl-2 and Bcl-xL on GP66-specific CD4 T cells from WT animals compared to OX40−/− mice (Fig. 7E). Collectively, these findings indicate that the failure of OX40-deficient effector T cells to accumulate in high numbers is a result of reduced survival of these cells, rather than an inability to divide sufficiently or secrete anti-viral or immunostimulatory cytokines.

## Discussion

Co-stimulation of T cells is a central component of adaptive immunity. Numerous co-stimulatory pathways have been described and it has become evident that the biological relevance of each of those pathways is greatly dependent on the immunologic context [36], e.g. it has been shown that signaling through the co-stimulatory CD27/CD70 pathway can impair T cell responses during persistent infection while it positively regulates T cells during acute infection [37], [38]. Thus, it is of great interest to define those co-stimulatory pathways that dominantly influence adaptive immunity in a given disease model. In order to do so, we chose to use the murine LCMV system as it offers the possibility to directly compare an acute viral infection that is effectively cleared within 7–10 days (LCMV Armstrong) to a protracted infection that persists for several weeks in various tissues (LCMV cl13). Our findings demonstrate that OX40-deficiency profoundly impacted the anti-viral immune response during persistent LCMV cl13 infection.

Although the role of OX40 has never been studied in a persistent viral infection with ongoing viral replication, much is known about the mechanisms by which OX40 can influence T cell responses. It has been demonstrated that OX40 promotes T cell survival, division and function in various immune models, including cancer models [39]–[41], viral [17], [18] and bacterial [31] infection as well as autoimmunity [42], [43]. Interestingly, during persistent infection, the role of OX40 seems to be largely restricted to promoting T cell survival, as we have not observed differences in T cell proliferation or cytokine production in the absence of OX40. In previous publications it has been shown that the ability of OX40 to promote T cell survival is based on its capacity to directly recruit and activate various intracellular signaling pathways, such as PI3K and Akt/PKB or NF-κB [33]–[35], [44]. These pathways control the expression of anti-apoptotic members of the Bcl-2 family and T cells that lack OX40 express reduced levels of Bcl-2, Bcl-xL, and Bfl-1 [14], [32]. Restoration of NF-κB activity or Akt activity through retroviral introduction of active IκB kinase or active Akt rescues survival and Bcl-2/Bcl-xL expression in OX40 deficient T cells [33]–[35]. We show here that OX40-deficient cells fail to accumulate, are more prone to apoptosis and display reduced levels of Bcl-2 and Bcl-xL, strongly suggesting that the OX40 induced signaling axis that controls the expression of anti-apoptotic molecules is functional in the context of persistent viral infection.

Although OX40 has primarily been associated with CD4 T cell function, it became evident in recent years that it can also strongly influence CD8 T cells [15], [17]. We observed much higher levels of OX40 expression on the anti-viral CD4 T cells compared to CD8 T cells, suggesting that OX40 might predominantly shape the CD4 response in this context. Consequently, the impaired CD8 T cell and B cell responses in the OX40-deficient mice could have been a result of insufficient CD4 T cell help as CD4 T cells are key players in providing help to both CD8 T cells and B cells. However, our adoptive cell-transfer model clearly demonstrates a crucial requirement of OX40 on both CD4 and CD8 T cells in order to accumulate sufficiently, implying direct OX40 signaling to CD8 T cells is likely important.

Another observation of our study that is noteworthy is how OX40 deficiency impacts the antiviral immune response already at early stages post infection, however, loss of viral control in those mice occurs in a delayed fashion. The notion that changes in the immune response can precede differences in viral titers in the LCMV cl13 system has been described before [4], [5], [45]. Indeed, even though anti-viral T cell numbers and LCMV-specific antibody-titers were markedly different as soon as day 10 and 20 post infection, this had no immediate impact on viral control and viral titers initially declined in both WT and OX40-deficient mice. Moreover, OX40-deficient mice had a clinical benefit from the weak immune response and displayed reduced signs of immunopathology. Thus, it seems that the amount of T cells that accumulates in WT mice in an OX40-dependent manner during early stages exceeds the number that is required for initial virus control. And while this over-abundance of pro-inflammatory effector T cells mediates immunopathology, it does enable stronger T cell pressure on viral replication over time, which eventually facilitates virus control.

While OX40 has previously been shown to positively regulate antigen-specific immune responses, the degree to which OX40 influences adaptive immunity and, importantly, facilitates virus control in the context of persistent infection is remarkable. Indeed, the strong impact of OX40 on the cellular and humoral immunity in the persistent LCMV-system is in contrast to previous findings in the acute LCMV system, where the absence of OX40 primarily affected the magnitude of the CD4 T cell response but did not have an impact on the antiviral CD8 T cell response, antibody titers and virus control [20]. Since our data suggest that OX40 expression is associated with antigen recognition, the rapid elimination of antigen in the acute infection model might be responsible for the loss of OX40 expression and thus, the reduction of biological relevance of OX40 signaling during acute infection. Those observations demonstrate different OX40 requirements for T cell accumulation during acute vs. persistent LCMV infection and, in addition, support our previous finding that OX40 usage may be dictated by the virulence of an invading pathogen [16].

While CD8 T cell responses have long been known to be critical for control of persistent LCMV-infection, the relevance of antibody responses in this context may have been underappreciated. Importantly, Pinschewer and colleagues showed that mice that were unable to produce LCMV-specific antibodies failed to control infection [46] and more recently, striking defects in control of LCMV cl13 were observed when antibody responses were reduced due to defective follicular T helper cell responses [5], [13]. These studies have established a strong role for T cell dependent humoral immunity in the control of persistent viruses and one study has demonstrated an important role of IL-6 in this context [13]. Our work, which we report in this manuscript, extends those observations and clearly establishes a critical role for OX40 in sustaining follicular T helper cell responses in the context of persistent viral infection.

Importantly, these findings could open new paths in the understanding of T cell failure during persistent viral infections in humans, i.e. chronic HCV infection. Particularly HCV-specific CD4 responses are very weak in persistently infected individuals [47], [48] and recently, it has been shown that CD4 T cell priming and accumulation occurs in the acute phase of HCV infection. However, in many cases, those CD4 responses rapidly disappear, severely increasing the risk of developing persistent viremia [12]. The rapid loss of CD4 responses in OX40 deficient mice is somewhat reminiscent of this observation, suggesting that OX40 might be an interesting molecule to study in those patients. The findings regarding the role of OX40 in the antibody response to a persisting virus might also be relevant to HIV infection in humans, as there is an intense interest in broadly neutralizing antibodies and why they only develop in a small subset of HIV-infected individuals [49], [50].

Collectively, our findings establish OX40 as a key factor in sustaining the cellular and humoral immunity during viral persistence and have important implications for the study of T cell dysfunction in persistent viral infections in humans.

## Materials and Methods

### Mice, virus and ethics statement

C57BL/6 and B6.SJL mice were purchased from The Jackson Laboratory and housed at the La Jolla Institute (LIAI) as well as OX40−/− mice on a C57BL/6 background [14]. LCMV-GP_61–80_-specific CD4 TCRtg (smtg) and LCMV-GP_33–41_-specific CD8 TCRtg mice (P14) were bred on an OX40−/− background. 5–8 week old, age and sex matched mice were infected intravenously with 2×10^6^ PFU of LCMV cl13 or intraperitoneally with 2×10^5^ PFU of LCMV Armstrong. All animal experiments were performed in strict accordance with the PHS Policy on Humane Care and Use of Laboratory Animals of the National Institutes of Health. All procedures were approved by and comply with the regulations of the La Jolla Institute Animal Care Committee (AP152-MvH6).

### Flow cytometry

Surface staining was performed on splenic single cell suspension with fluorescently-labeled or biotinylated antibodies against CD4, CD8, CD19, B220, CD150 (SLAM), CXCR5, PD-1, KLRG1, FAS, IgD, CD138, CD45.1, CD45.2, OX40L and fluorescently-labeled PNA as well as GP33-pentamers and GP66-tetramers. Streptavidin- APC and -PE were used to stain biotinylated antibodies. Bcl6, Bcl-2, Bcl-xL and active caspase 3 were stained intracellularly after permeabilization with Cytofix/Cytoperm (BD). For most experiments, LIVE/DEAD (Invitrogen) viability dye was used to exclude dead cells.

For ICCS, cells were stimulated with CD8- (GP33, GP276) and CD4-restricted (GP61) LCMV-epitopes (10 µg/ml) for five hours at 37°C in supplemented RPMI-medium (Invitrogen). Cells were permeabilized using Cytofix/Cytoperm (BD) and stained with fluorescently-labeled antibodies against IL-2, IFN-γ, and TNF. IL-21 staining was performed as described previously [51] using an IL-21R-FC chimeric protein (R&D Systems) and a PE-labeled anti-human IgG (Jackson Immunoresearch). All samples were acquired on a LSRII (BD) using DIVA software (BD) and analyzed using FlowJo software (Tree Star). Antibodies were obtained from BD, BioLegend or eBioscience.

### Cell transfer

Spleens were harvested from TCRtg mice (smtg or P14) on a WT and OX40−/− background. CD4 (smtg) and CD8 (P14) isolation was performed using purified rat antibodies against B220, CD11c, CD11b, CD16/32, I-A/I-E and CD4 or CD8 and sheep anti-rat Dynabeads (Invitrogen). For co-transfer experiments into the same or separate hosts, equal numbers of OX40+/+(WT), OX40+/−(Het) and/or OX40−/−(KO) cells (2,000 P14, 5,000 smtg; 1,000,000 for d3 analysis) were transferred into CD45.1/2 mismatched recipients. Cells were labeled with CFSE (BioChemika) prior to transfer in selected experiments. For analysis of proliferation at later time points we used the BrdU-Proliferation kit from BD.

### Plaque assay

Monolayers of Vero cells (ATCC #CRL-1587) were exposed to tissue homogenate or serum of individual mice in 1/10 dilutions. Plaque counts were performed 5 (organs) or 6 (serum) days post infection. Vero cells were cultured and maintained in supplemented DMEM (Invitrogen) at 37°C.

### ELISA

Elisa plates (Nunc) were coated overnight with LCMV infected cell lysate. LCMV-specific IgG was detected in serum samples using HRP goat anti-mouse IgG (Invitrogen). SureBlue Reserve TMB Kit (KPL) functioned as substrate. Absorbance was detected by a Spectra Max M2*^e^* (Molecular Devices).

### Statistical analysis

Statistical analyses were performed and line art figures were designed using GraphPad Prism 5 software (GraphPad). If not specified otherwise, the unpaired, two tailed Student's t-test was used. For viral loads, the log rank test was applied with a defined endpoint of viral titers below the limit of detection. All error bars are SD. Values of p<0.05 were considered significant. *p<0.05, **p<0.01 and ***p<0.001.
